# Altered insula–default mode network connectivity in fibromyalgia: a resting-state magnetoencephalographic study

**DOI:** 10.1186/s10194-017-0799-x

**Published:** 2017-08-23

**Authors:** Fu-Jung Hsiao, Shuu-Jiun Wang, Yung-Yang Lin, Jong-Ling Fuh, Yu-Chieh Ko, Pei-Ning Wang, Wei-Ta Chen

**Affiliations:** 1 Brain Research Center, National Yang-Ming University, Taipei, Taiwan; 2 Institute of Brain Science, National Yang-Ming University, Taipei, Taiwan; 3 School of Medicine, National Yang-Ming University, Taipei, Taiwan; 4 Neurological Institute, Taipei Veterans General Hospital, No. 201, Sec. 2 Shih-Pai Rd, Taipei, Taiwan; 5 Department of Ophthalmology, Taipei Veterans General Hospital, Taipei, Taiwan

**Keywords:** Fibromyalgia, Insula, Default mode network, Resting state, Functional connectivity, Magnetoencephalography (MEG)

## Abstract

**Background:**

Fibromyalgia (FM) is a disabling chronic pain syndrome with unknown pathophysiology. Functional magnetic resonance imaging studies on FM have suggested altered brain connectivity between the insula and the default mode network (DMN). However, this connectivity change has not been characterized through direct neural signals for exploring the embedded spectrotemporal features and the pertinent clinical relevance.

**Methods:**

We recorded the resting-state magnetoencephalographic activities of 28 patients with FM and 28 age- and sex-matched controls, and analyzed the source-based functional connectivity between the insula and the DMN at 1–40 Hz by using the minimum norm estimates and imaginary coherence methods. We also measured the connectivity between the DMN and the primary visual (V1) and somatosensory (S1) cortices as intrapatient negative controls. Connectivity measurement was further correlated with the clinical parameters of FM.

**Results:**

Compared with the controls, patients with FM reported more tender points (15.2±2.0 vs. 5.9±3.7) and higher total tenderness score (TTS; 29.1±7.0 vs. 7.7±5.5; both *p* < 0.001); they also had decreased insula–DMN connectivity at the theta band (4–8 Hz; left, *p* = 0.007; right, *p* = 0.035), but displayed unchanged V1–DMN and S1–DMN connectivity (*p* > 0.05). When patients with FM and the controls were combined together, the insula-DMN theta connectivity was negatively correlated with the number of tender points (left insula, *r* = −0.428, *p* = 0.001; right insula, *r* = −0.4, *p* = 0.002) and TTS score (left insula, *r* = −0.429, *p* = 0.001; right insula, *r* = −0.389, *p* = 0.003). Furthermore, in patients with FM, the right insula–DMN connectivity at the beta band (13–25 Hz) was negatively correlated with the number of tender points (*r* = −0.532, *p* = 0.004) and TTS (*r* = −0.428, *p* = 0.023), and the bilateral insula–DMN connectivity at the delta band (1–4 Hz) was negatively correlated with FM Symptom Severity (left: *r* = −0.423, *p* = 0.025; right: *r* = −0.437, *p* = 0.020) and functional disability (Fibromyalgia Impact Questionnaire; left: *r* = −0.415, *p* = 0.028; right: *r* = −0.374, *p* = 0.050).

**Conclusions:**

We confirmed the frequency-specific reorganization of the insula–DMN connectivity in FM. The clinical relevance of this connectivity change may warrant future studies to elucidate its causal relationship and potential as a neurological signature for FM.

**Electronic supplementary material:**

The online version of this article (doi:10.1186/s10194-017-0799-x) contains supplementary material, which is available to authorized users.

## Background

Fibromyalgia (FM) is a common chronic pain disorder, with a prevalence of 2–4% in the general population [1]. FM is characterized by chronic, widespread pain along with various clinical symptoms that reflect a centralized pain state, including fatigue, insomnia, cognitive dysfunction, headache, and depression [2]. Because of its polysymptomatic nature, the prognosis of FM is highly distressful for the patients and cost intensive for the society [1].

The pathophysiology of FM remains unclear. Patients with FM are hypersensitive to painful and nonpainful stimuli and exhibit increased brain responses within the so-called “pain network,” including the insular cortex, anterior cingulate cortex (ACC), primary (S1) and secondary somatosensory cortices, and thalamus [3–5]. Functional magnetic resonance imaging (fMRI) studies on FM have shown brain connectivity changes in this pain network, along with some intrinsic connectivity networks that exhibited synchronous activity in the resting state (i.e., resting-state networks), such as the default mode network (DMN) [6, 7], salience network [8], and sensorimotor network [9]. Taken together, the chronic and persistent pain of FM appears to change a patient’s brain responses during the processing of both externally driven information and internally generated thoughts.

The DMN comprises a set of synchronous brain regions such as the medial prefrontal and posterior cingulate cortices that are active at rest and deactivated during task performance [10]. This resting-state network can modulate pain perception through autonomic and antinociceptive descending modulation networks [11]. A recent meta-analysis of voxel-based morphometry studies indicated that patients with FM exhibit gray matter atrophy in the left medial prefrontal and right dorsal posterior cingulate cortices, both of which are key regions within the DMN [12]. Moreover, the DMN is disrupted in the activation–deactivation dynamics in the presence of chronic pain, suggesting that the DMN is a primary resting-state network affected by chronic pain [13]. Thus far, the mechanism underlying the interaction between the DMN and pain network brain regions in the context of chronic pain remains largely unknown [14]. Fallon et al. have used fMRI to investigate the blood-oxygen-level-dependent (BOLD) signal fluctuations in the DMN structures of patients with FM and demonstrated altered connectivity with various regions associated with pain, cognitive and emotional processing [15]. In other fMRI studies, Napadow and colleagues [7] reported increased connectivity between the DMN and insula, a pivotal region of the pain network involved in multidimensional (sensory, affective, and cognitive) pain processing [16–18]. Notably, the insula–DMN connectivity was correlated with the individual level of spontaneous pain reported at the time of scanning, and it presented a corresponding decrease after the alleviation of pain following pregabalin treatment [19] or acupuncture intervention [6]. This altered insula–DMN connectivity could not be confirmed by another fMRI study on FM [9]; nevertheless, corroborating evidence of elevated coupling between the DMN and insula has been noted for other pain disorders, such as chronic back pain [20], diabetic neuropathic pain [21], and acute migraine headache [22].

Given its potential to encode clinical pain and serve as an objective measure of FM phenotypes, further characterization of the insula–DMN connectivity is rucial. The DMN and insula are critically involved in pain perception and both structures may present correlated activities in a variety of tasks such as attention or self-recognition [14, 23, 24]. A previous resting-state fMRI study in FM also reported an association between individual ratings of pain sensitivity and the insula connectivity with midline regions of the DMN (posterior cingulate and medial prefrontal cortices) [9]. Thus, characterization of the insula-DMN connectivity may enhance our understanding towards the pathophysiology of FM.

To date, most of the related fMRI studies in insula-DMN connectivity have measured very-low-frequency (<0.1 Hz) fluctuations of resting-state BOLD signals rather than directly recording neural oscillatory changes through a spectrotemporal analysis of a wide-frequency domain. However, chronic pain potentially changes the dynamic brain activities at specific frequency bands of >0.1 Hz [21, 25]. Some electroencephalography (EEG) studies on FM have shown spectral power changes at higher brain oscillation frequency bands (>1 Hz), especially within the theta range (4–8 Hz) [26–28], but pertinent analysis for the connectivity change between specific brain regions remain inadequate, probably because of the constraint of EEG spatial resolution. Therefore, this study investigated the resting-state functional connectivity pattern between the DMN and insula across different frequency bands through magnetoencephalography (MEG), which enables the visualization of explicit neural oscillatory features, similar to traditional EEG but with finer spatial localization [29]. MEG has been used to characterize brain oscillatory changes in various chronic pain conditions, including chronic migraine [30], phantom limb pain [31] and complex regional pain syndrome [32]. In FM, a resting-state MEG study reported increased theta, beta and gamma oscillations in the prefrontal cortex [33]. We hypothesized that the insula–DMN connectivity is altered by the chronic and persistent pain perception of FM, possibly reflecting the clinical phenotype of FM in a frequency-dependent manner.

## Methods

### Patients

Consecutive patients with FM aged 20–60 years were enrolled from the Neurological Institute of Taipei Veterans General Hospital in Taiwan. All patients fulfilled the modified 2010 American College of Rheumatology (ACR) Fibromyalgia Diagnostic Criteria [2]; however, those with any autoimmune rheumatic disease were excluded. Healthy age- and sex-matched volunteers who did not have past or family histories of FM and who had not experienced any significant pain during the past year were recruited as the controls. All participants were right-handed, denied having any history of systemic or major neuropsychiatric disease, and had normal physical and neurological examination results as well as normal brain MRI results. Participants who were receiving any medication or hormone therapy on a daily basis were excluded. To minimize the effects of hormones on results, MEG was administered to women of reproductive age only during the luteal phase, as estimated by their last menstrual cycle and confirmed by their next menstruation through telephone interviews. The hospital’s institutional review board approved the study protocol, and each participant provided written informed consent.

Immediately before undergoing MEG, all patients with FM completed a questionnaire on the distribution (Widespread Pain Index [2]), intensity (0–10 on a numerical rating scale), and duration (years) of their pain and the accompanying somatic or psychiatric symptoms, including fatigue, unrefreshing sleep, cognitive symptoms, headache, lower abdominal pain or cramps, and depression (Symptom Severity Scale [2]). The revised Fibromyalgia Impact Questionnaire (FIQR) was administered to the patients with FM to assess their FM-related functional disability [34]. To evaluate anxiety and depression severity, the Hospital Anxiety and Depression Scale (HADS) was administered to all participants [35]. Each participant also completed a manual tender-point survey on the 18 specific anatomical positions defined by the 1990 ACR FM classification [36]. In response to direct palpation with a dolorimeter at a 4.0-kg/m^2^ force level, each participant reported the level of tenderness (0: none; 1: mild; 2: moderate; 3: severe) at each position. We determined the number of tender points (range, 0–18) and the total tenderness score (TTS = sum of tenderness level in the 18 positions; range 0–54) in each participant. The number of tender points in patients with FM has been found to be associated with FM-related variables (pain, fatigue, sleep, anxiety, depression, and global severity) and the rheumatology distress index, a composite measure of distress constructed from scores of sleep disturbance, fatigue, anxiety, depression, and global severity [37].

### MEG recording

MEG data were obtained using a whole-scalp 306-channel neuromagnetometer (Vectorview; Elekta Neuromag, Helsinki, Finland), which is composed of 102 identical triple sensor elements; each sensor element comprises one magnetometer and two orthogonal planar gradiometers. The exact position of the head with respect to the sensors was obtained by measuring magnetic signals produced by current leads to four head indicator coils at known sites on the scalp. Individual Cartesian coordinates were then determined using a three dimensional (3D) digitizer. To obtain precise registration, approximately 50 additional scalp points were also digitized. These landmarks of the head position enabled further alignment of the MEG and magnetic resonance (MR) imaging coordinate systems.

We began the MEG recording with a 3-min empty-room recording to capture sensor and environmental noise; the data were then applied to calculate the noise covariance in a subsequent source analysis. During the resting-state recording, participants sat comfortably with their head supported by the helmet of the neuromagnetometer. They were asked to close their eyes, but remain awake, relaxed, and not perform any explicit task. Cortical spontaneous activity data were collected for 3 min and digitized at 600 Hz. The recording was repeated if a participant fell asleep or had excessive within-run head movement (displacement >5 mm in the x, y, or z plane of the head position indicator). Electrooculography and electrocardiography were performed simultaneously.

### Data preprocessing

MEG data can become contaminated because of patient-related or environmental factors. Therefore, we visually inspected all data for segments the containing artifacts from head movement or environmental noise and discarded contaminated segments. To remove powerline contaminations, notch filters (60 Hz and its harmonics) were used. Moreover, Brainstorm’s ECG and EOG detection functionality [38] automatically identified heartbeat and eye blinking events; for these event data segments, projectors were defined through principal component analysis separately. The principal components meeting the artifact’s sensor topography were then manually selected and excluded through orthogonal projection [39]. Individual brain MR images were acquired by using a 3 T MR system (Siemens Magnetom Tim Trio, Erlangen, Germany), with a TR of 9.4 ms, TE of 4 ms, recording matrix of 256 × 256 pixels, field of view of 256 mm, and slice thickness of 1 mm. The surface model was automatically reconstructed from the T1-weighted structural volumetric images (BrainVISA 4.5.0, http://brainvisa.info). The detailed geometric reconstruction of the scalp, brain gray and white matter, and tessellations used to estimate the gray and white matter border provided a topographical 3D representation of the brain surface.

### Data analysis

We analyzed resting-state functional connectivity in two stages:

In the first part, the resting-state MEG recording of each participant was analyzed using depth-weighted minimum norm estimates to obtain the distributed and dynamic cortical source model [40], which presented each cortical vertex as a current dipole and included ~15,000 vertices in the forward model. The cortical source model of each participant was then morphed into a common source space defined by the Colin27 anatomy [41]. For the connectivity analysis, we defined regions of interest (ROIs) in the structural T1 template volume by using Mindboggle cortical parcellation [42], and selected 12 DMN-related brain regions based on previous studies [10, 23, 43], including the bilateral posterior cingulate cortex, precuneus, inferior parietal cortex, medial temporal cortex, medial frontal cortex, and lateral temporal regions. The time-varying source density of each ROI was obtained from the averaged source activities of each vertex in the ROI. Although this study focused on changes in the insula–DMN connectivity between groups, the connectivity of the DMN with S1 and V1 was also investigated to strengthen the specificity of our findings. S1 is a sensory-discriminative region of the pain network, whereas V1 belongs to the medial visual network and generally unrelated to pain information processing. An fMRI study on FM reported that both S1 and V1 do not exhibit an altered resting-state functional connectivity with the DMN [7]. The methodology of this part has been published in our recent studies elsewhere [44–46].

The second part involved the analysis of functional connectivity from the time-varying source density of each ROI by using of the imaginary coherence method, which essentially measures how the phases between two sources are coupled to each other with minimum crosstalk effects between sources [47]. This technique can effectively reveal altered functional connectivity in patients with brain lesions [48], brain tumors [49], and Alzheimer disease [50] during the resting-state condition. Moreover, this technique rejects the spurious connectivity between two cortical sources without time delay, which could be attributed to a common source or volume conduction. Thus, imaginary coherence represents the interactions between brain regions with a specific time lag [47]. In the present study, we computed the imaginary coherence values between the 12 DMN regions and the bilateral insula, S1, and V1 by using the FieldTrip toolbox (http://fieldtrip.fcdonders.nl/) and obtained the full 6×12 adjacency matrix. Then, the node strengths (the sum of IC values connected to the node) of the 6 regions (bilateral insula, S1, and V1) were individually estimated to represent the bilateral insula–DMN, S1–DMN, and V1–DMN functional connectivity, respectively. The results were categorized by frequency bands: delta (1–4 Hz), theta (4–8 Hz), alpha (8–13 Hz), beta (13–25 Hz), and gamma (25–40 Hz).

### Statistical analysis

The demographics and clinical profiles of the control and FM groups were compared using the Student *t* or chi-square test, as appropriate. The group differences in the insula–DMN, S1–DMN, and V1–DMN connectivity at each frequency band were also examined using analysis of covariance, with age, sex, anxiety (HADS anxiety score), and depression (HADS depression score) regressed out as covariates of no interest. Pearson’s correlation was used to determine the correlation between MEG connectivity measures and clinical FM profiles. The clinical correlations were further verified using multiple regression analysis to adjust for age, sex, anxiety, and depression effects. Throughout the statistical analyses, the Bonferroni correction was used for multiple comparisons and a *p* value of <0.05 was considered statistically significant.

## Results

### Demographics and clinical profiles

The demographics and clinical profiles of patients with FM and the controls are summarized in Table 1. Age and sex did not differ between the groups. However, compared with the controls, patients with FM reported more tender points and had higher TTS and HADS scores (all *p* < 0.001).

**Table 1:** Demographic and clinical profiles of patients with fibromyalgia (FM) and controls

	Groups		*P* value
	Control	FM	
Subject number (n)	28	28	-
Age (years)	42.5±7.6	42.1±10.6	0.886
Sex	26F/2M	26F/2M	-
HADS total score (0–42)	9.6±6.1	20.3±6.5	<0.001^*^
HADS anxiety score (0–21)	4.6±3.4	11.6±3.5	<0.001^*^ <0.001^*^
HADS depression score (0–21)	3.6±3.1	8.7±3.8	<0.001^*^
Number of tender points (0–18)	5.9±3.7	15.2±2.0	<0.001^*^
Total Tenderness Score (0–54)	7.7±5.5	29.1±7.0	<0.001^*^
Clinical pain intensity (0–10)	-	5.28±2.4	-
Disease duration (years)	-	7.3±9.2	-
Widespread Pain Index (0–19)	-	9.9±4.3	-
Symptom Severity (0–12)	-	7.9±1.7	-
Revised Fibromyalgia Impact Questionnaire (0–100)	-	50.6±20.6	-

*HADS* the hospital anxiety and depression scale

^*^ *p* < 0.001

### Altered insula–DMN connectivity in FM

**Fig. 1:**
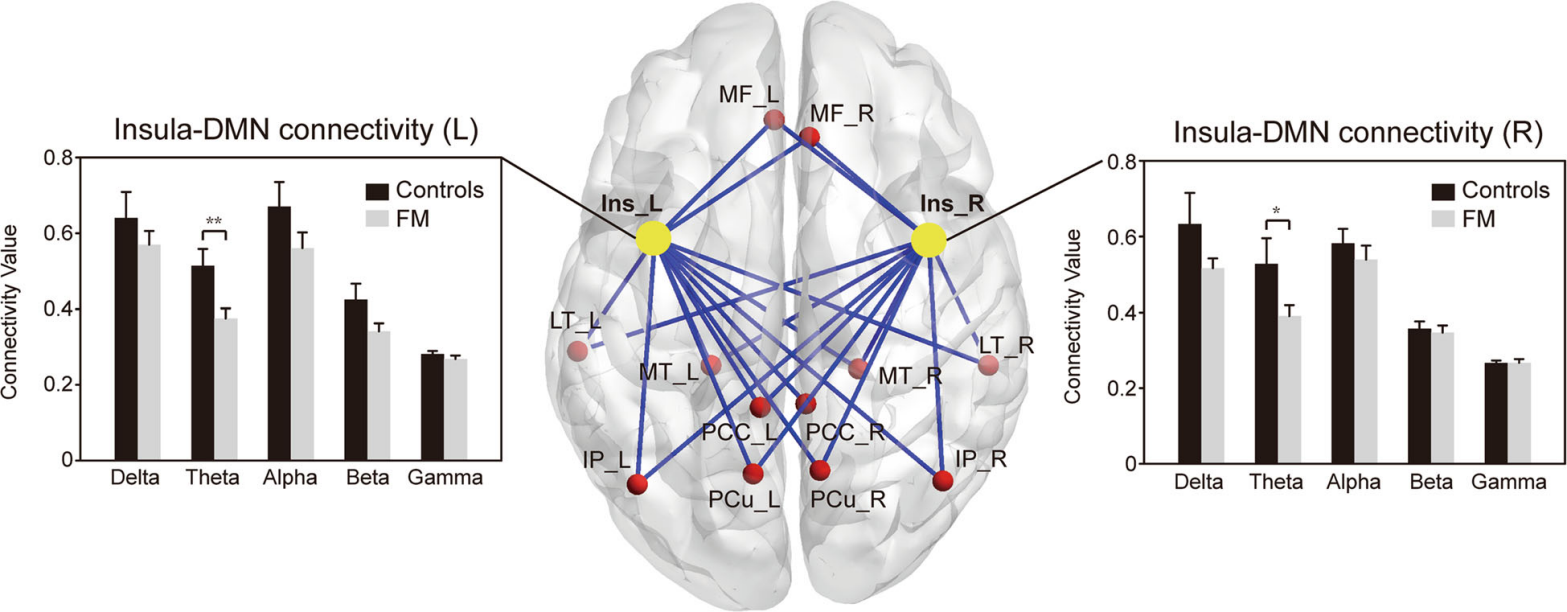
Functional connectivity between the insula (Ins) and default mode network (DMN) at the delta (1–4 Hz), theta (4–8 Hz), alpha (8–13 Hz), beta (13–25 Hz), and gamma (25–40 Hz) bands, assessed using resting-state magnetoencephalography brain activity and compared between controls and patients with fibromyalgia (FM). L, left; R, right; MF, medial frontal; LT, lateral temporal; MT, medial temporal; PCC, posterior cingulate cortex; IP, inferior parietal; PCu, precuneus. *, *p* < 0.05; **, *p* < 0.01

In general, the bilateral insula–DMN connectivity tended to be decreased at all frequency bands in patients with FM compared with the controls (Fig. 1); however, the difference was significant only at the theta band on the left (*F*(1,54) = 7.975, *p* = 0.007) and right (*F*(1,54) = 4.719, *p* = 0.035) sides. The decreased theta connectivity between insula and each area of the DMN is shown in Additional file 1: Table S1 and Additional file 2: Table S2. By contrast, no difference was noted in the bilateral S1–DMN or V1–DMN connectivity at any of the frequency bands (all *p* > 0.1).

### Clinical correlation

**Fig. 2:**
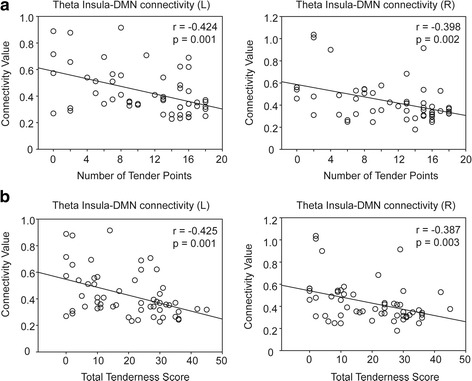
Correlation of the insula–default mode network (DMN) connectivity at the theta band (4–8 Hz) with the (**a**) number of tender points (0–18) and (**b**) total tenderness scores (0–54) in all participants (patients with FM and the controls)

**Fig. 3:**
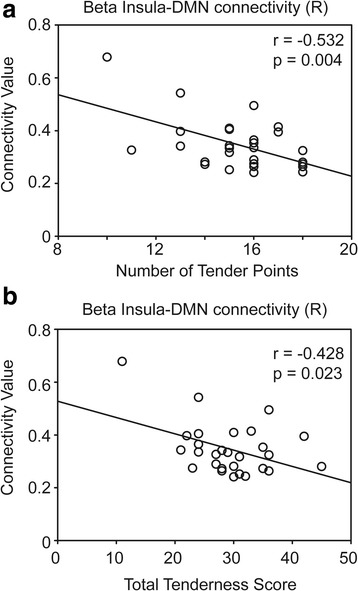
Correlation of the right insula–default mode network (DMN) connectivity at the beta band (13–25 Hz) with the (**a**) number of tender points (0–18) and (**b**) total tenderness scores (0–54) of patients with FM

**Fig. 4:**
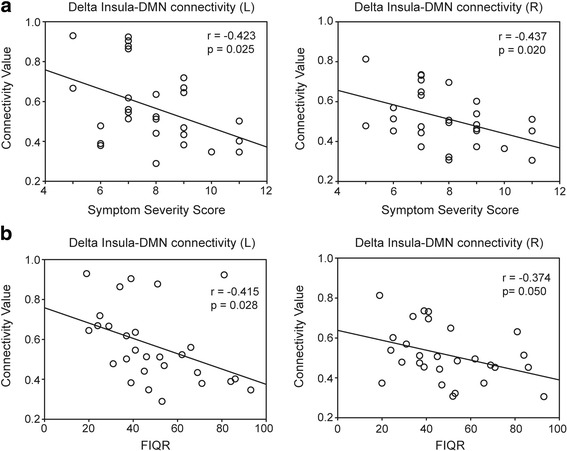
Correlation of insula–default mode network (DMN) connectivity at the delta band (1–4 Hz) with the (**a**) Symptom Severity Scale (0–12) and (**b**) revised Fibromyalgia Impact Questionnaire (FIQR; 0–100) scores

In all participants, the theta connectivity was negatively correlated with the number of tender points (left insula, *r* = −0.428, *p* = 0.001; right insula, *r* = −0.4, *p* = 0.002) and the TTS (left insula, *r* = −0.429, *p* = 0.001; right insula, *r* = −0.389, *p* = 0.003) (Fig. 2). In patients with FM, the right insula–DMN connectivity at the beta band also showed a negative correlation with the number of tender points (*r* = −0.532, *p* = 0.004) and the TTS (*r* = −0.428, *p* = 0.023; Fig. 3). In addition, the bilateral insula–DMN connectivity at the delta band was negatively correlated with Symptom Severity Scale (left: *r* = −0.423, *p* = 0.025; right: *r* = −0.437, *p* = 0.020) or the degree of functional impairment (FIQR; left: *r* = −0.415, *p* = 0.028; right: *r* = −0.374, *p* = 0.05; Fig. 4).

No clinical correlation of the insula–DMN connectivity was noted at the alpha or gamma band. Moreover, the clinical pain intensity and duration of FM were not correlated with any MEG connectivity measure (all *p* > 0.05).

Adjustment for the individual differences in age, sex, anxiety, and depression during multiple regression analyses did not change the aforementioned clinical correlation results.

## Discussion

The main finding of this study is that patients with FM had decreased resting-state bilateral insula–DMN connectivity at the theta band. When patients with FM and the controls were examined together, the insula-DMN theta connectivity was negatively correlated with tenderness. Moreover, in patients with FM, the insula–DMN connectivity was also negatively correlated with tenderness at the beta band and with centralized pain-related symptoms (Symptom Severity Scale) and functional impairment (FIQR) at the delta band.

Studies have reported the existence of intrinsic connectivity between the insula and DMN in healthy individuals [16, 51]. During pain processing, the insula has been proposed to serve as a switching core that relays sensory information into higher-order affective and cognitive modulation [16–18], whereas the DMN has been linked to pain modulation through descending inhibitory pathways [11]. Thus, the present finding regarding disrupted insula–DMN connectivity may implicate impaired pain modulation leading to the chronic pain of FM. Similarly, MRI studies on FM have reported that the DMN regions had decreased gray matter volume [52, 53] and functional connectivity with specific regions of the pain network [54, 55]. Moreover, a quantitative EEG study on FM demonstrated widespread hypocoherence in the frontal brain regions [28]. Overall, these overlapping brain changes may reflect the central sensitization mechanism underlying FM [56].

The present finding of disrupted insula–DMN connectivity in FM appears to contrast with previous fMRI results demonstrating increased coupling of the BOLD signals between the insula and DMN [6, 7]. However, the discrepancy in the altered connectivity patterns could be explained by the fundamental methodological differences between fMRI and MEG [57–59], the frequency-dependent oscillatory characteristics of the underlying neural network [45, 60], and the effects of autonomic regulation on BOLD responses [61], which may be impaired in patients with FM [62, 63]. All of these apparently contradictory connectivity changes at different frequencies potentially characterize a common functional reorganization mechanism in FM. In agreement with this, several studies using different modalities to characterize the brain oscillatory change in the same disease have yielded contrasting patterns of connectivity change [45, 60, 61]. The present finding of altered insula–DMN connectivity in FM is further supported by the similarity in the findings of bilateral insula-individual DMN areas (Additional file 1: Table S1 and Additional file 2: Table S2), absence of changes in V1–DMN or S1–DMN connectivity, and clinical relevance.

Our results demonstrate that the insula–DMN connectivity in FM was significantly decreased at the theta band. A recent review identified the theta oscillation as the main change that occurs in brain rhythm during chronic pain [64]. A quantitative EEG study that included patients with FM showed widespread hypocoherence in the frontal brain regions at low to middle frequencies, including the theta band [28]. In line, recent resting EEG and MEG studies in FM also showed altered theta oscillations in midline brain structure such as medial prefrontal cortex [26, 33]. Theta oscillation has been linked to working memory, attention, emotional arousal, and fear conditioning, all of which may be related to pain processing [65, 66]. Moreover, theta connectivity at the bilateral insula cortex has been reported to be correlated with pain perception. In a 64-channel EEG study using electrical stimulation at the threshold level, trials perceived as painful were characterized by a lower prestimulus theta connectivity, compared with trials rated as nonpainful [67]. We also found a negative correlation between tenderness and insular-DMN theta connectivity. Thus, the present findings of decreased theta connectivity between the insula and the DMN may reflect persistent pain encoding associated with the chronic pain state of FM.

Despite its lack of correlation with clinical pain intensity, we also noted that the insula–DMN connectivity was negatively correlated with tenderness at the beta band in patients with FM. In pain processing, the beta oscillation is associated with top-down attention modulation [68, 69] and the perceptual integration of sensory and contextual (cognitive, emotional, and motivational) information [64, 70]. Therefore, the increased tenderness in patients with FM may be justified by an inefficient attentional modulation or impaired recruitment of contextually appropriate brain networks, resulting in the widespread body pain phenotype. By contrast, the insula–DMN connectivity at the delta frequency was negatively correlated with centralized pain-related symptoms (Symptom Severity scale) and functional impairment (FIQR) in patients with FM. The delta oscillation has been suggested to be the neuropathological hallmark of brain rhythm in mood disorders [71, 72], cognitive impairment [50, 73], pain attack [74], and fatigue [75, 76]. Therefore, our present findings highlight the complex role of neural synchrony between the insula and the DMN in pain, emotional, and cognitive processing, as shown previously [16–18]. Future studies should elucidate whether the delta synchrony of the insula–DMN network serves as the common neural basis for the polysymptomatic nosology and multidomain functional disability in patients with FM.

This study has several limitations. First, the anterior and posterior insula have been reported to be functionally segregated regions with different connectivity [16–18]; however, we could not differentiate these subregions because of the constraint of the Colin27 anatomical labeling template. Nevertheless, the altered insula–DMN connectivity in FM has been shown to involve both the anterior and posterior insula [7]. Second, a prior study proposed that no one-to-one correspondence occurs between any frequency component of brain activity and pain [77]. Notably, brain activity at different frequencies provides different and complementary information regarding pain, and the relationship between pain and brain activity may be variable and context dependent [64]. Thus, the present findings should be interpreted with caution when being generalized to other clinical contexts or pain disorders. Limited by its cross-sectional design, this study could not clarify the causal relationship of the connectivity changes in FM. Although we did not observe a clinical correlation between connectivity measures and disease duration (favoring the present findings as the consequences of FM), additional confirmatory longitudinal studies are warranted. Finally, the present finding of connectivity change may be problematic if such change is confounded by the ongoing pain perceived in patients with FM, despite a lack of correlation between the connectivity changes and the clinical pain intensity. Additional longitudinal studies controlling the intrasubject pain variation may therefore help re-confirm the “true” resting-state connectivity change.

## Conclusion

The insula–DMN connectivity is associated with frequency-specific functional reorganization in patients with FM. The clinical relevance of this connectivity change may provide an objective measure of FM phenotypes and related functional disability. However, the confirmation of its causal relationship and potential as a neurological signature for FM requires further research.

## Additional files

Additional file 1: Table S1. Functional connectivity between the left insula and default mode network (12 cortical areas) in healthy controls and patients with fibromyalgia. (DOCX 20 kb)

Additional file 2: Table S2. Functional connectivity between the right insula and default mode network (12 cortical areas) in healthy controls and patients with fibromyalgia. (DOCX 22 kb)

## References

[1] Hauser W, Ablin J, Fitzcharles MA, Littlejohn G, Luciano JV, Usui C, Walitt B (2015). Fibromyalgia. Nat Rev Dis Primers.

[2] Wolfe F, Clauw DJ, Fitzcharles MA, Goldenberg DL, Hauser W, Katz RS, Mease P, Russell AS, Russell IJ, Winfield JB (2011). Fibromyalgia criteria and severity scales for clinical and epidemiological studies: a modification of the ACR preliminary diagnostic criteria for fibromyalgia. J Rheumatol.

[3] Cook DB, Lange G, Ciccone DS, Liu WC, Steffener J, Natelson BH (2004). Functional imaging of pain in patients with primary fibromyalgia. J Rheumatol.

[4] Gracely RH, Petzke F, Wolf JM, Clauw DJ (2002). Functional magnetic resonance imaging evidence of augmented pain processing in fibromyalgia. Arthritis Rheum.

[5] Lopez-Sola M, Pujol J, Wager TD, Garcia-Fontanals A, Blanco-Hinojo L, Garcia-Blanco S, Poca-Dias V, Harrison BJ, Contreras-Rodriguez O, Monfort J, Garcia-Fructuoso F, Deus J (2014). Altered functional magnetic resonance imaging responses to nonpainful sensory stimulation in fibromyalgia patients. Arthritis Rheumatol.

[6] Napadow V, Kim J, Clauw DJ, Harris RE (2012). Decreased intrinsic brain connectivity is associated with reduced clinical pain in fibromyalgia. Arthritis Rheum.

[7] Napadow V, LaCount L, Park K, As-Sanie S, Clauw DJ, Harris RE (2010). Intrinsic brain connectivity in fibromyalgia is associated with chronic pain intensity. Arthritis Rheum.

[8] Ichesco E, Schmidt-Wilcke T, Bhavsar R, Clauw DJ, Peltier SJ, Kim J, Napadow V, Hampson JP, Kairys AE, Williams DA, Harris RE (2014). Altered resting state connectivity of the insular cortex in individuals with fibromyalgia. J Pain.

[9] Flodin P, Martinsen S, Lofgren M, Bileviciute-Ljungar I, Kosek E, Fransson P (2014). Fibromyalgia is associated with decreased connectivity between pain- and sensorimotor brain areas. Brain Connect.

[10] Raichle ME, MacLeod AM, Snyder AZ, Powers WJ, Gusnard DA, Shulman GL (2001). A default mode of brain function. Proc Natl Acad Sci U S A.

[11] Kucyi A, Salomons TV, Davis KD (2013). Mind wandering away from pain dynamically engages antinociceptive and default mode brain networks. Proc Natl Acad Sci U S A.

[12] Lin C, Lee SH, Weng HH (2016). Gray matter atrophy within the default mode network of fibromyalgia: a meta-analysis of Voxel-based Morphometry studies. Biomed Res Int.

[13] Baliki MN, Geha PY, Apkarian AV, Chialvo DR (2008). Beyond feeling: chronic pain hurts the brain, disrupting the default-mode network dynamics. J Neurosci.

[14] Farmer MA, Baliki MN, Apkarian AV (2012). A dynamic network perspective of chronic pain. Neurosci Lett.

[15] Fallon N, Chiu Y, Nurmikko T, Stancak A (2016). Functional connectivity with the default mode network is altered in fibromyalgia patients. PLoS One.

[16] Cauda F, D'Agata F, Sacco K, Duca S, Geminiani G, Vercelli A (2011). Functional connectivity of the insula in the resting brain. NeuroImage.

[17] Chang LJ, Yarkoni T, Khaw MW, Sanfey AG (2013). Decoding the role of the insula in human cognition: functional parcellation and large-scale reverse inference. Cereb Cortex.

[18] Deen B, Pitskel NB, Pelphrey KA (2011). Three systems of insular functional connectivity identified with cluster analysis. Cereb Cortex.

[19] Harris RE, Napadow V, Huggins JP, Pauer L, Kim J, Hampson J, Sundgren PC, Foerster B, Petrou M, Schmidt-Wilcke T, Clauw DJ (2013). Pregabalin rectifies aberrant brain chemistry, connectivity, and functional response in chronic pain patients. Anesthesiology.

[20] Loggia ML, Kim J, Gollub RL, Vangel MG, Kirsch I, Kong J, Wasan AD, Napadow V (2013). Default mode network connectivity encodes clinical pain: an arterial spin labeling study. Pain.

[21] Cauda F, Sacco K, Duca S, Cocito D, D'Agata F, Geminiani GC, Canavero S (2009). Altered resting state in diabetic neuropathic pain. PLoS One.

[22] Coppola G, Di Renzo A, Tinelli E, Di Lorenzo C, Scapeccia M, Parisi V, Serrao M, Evangelista M, Ambrosini A, Colonnese C, Schoenen J, Pierelli F (2017) Resting state connectivity between default mode network and insula encodes acute migraine headache. Cephalalgia. doi:10.1177/0333102417715230 10.1177/0333102417715230 28605972

[23] Buckner RL, Andrews-Hanna JR, Schacter DL (2008). The brain's default network: anatomy, function, and relevance to disease. Ann N Y Acad Sci.

[24] Craig AD (2009). How do you feel--now? The anterior insula and human awareness. Nat Rev Neurosci.

[25] Malinen S, Vartiainen N, Hlushchuk Y, Koskinen M, Ramkumar P, Forss N, Kalso E, Hari R (2010). Aberrant temporal and spatial brain activity during rest in patients with chronic pain. Proc Natl Acad Sci U S A.

[26] Fallon N, Chiu Y, Nurmikko T, Stancak A (2017) Altered theta oscillations in resting EEG of fibromyalgia syndrome patients. Eur J Pain. doi:10.1002/ejp.1076 10.1002/ejp.1076 PMC5763419 28758313

[27] Gonzalez-Roldan AM, Cifre I, Sitges C, Montoya P (2016) Altered dynamic of EEG oscillations in fibromyalgia patients at rest. Pain Med. doi:10.1093/pm/pnw023 10.1093/pm/pnw023 26921889

[28] Hargrove JB, Bennett RM, Simons DG, Smith SJ, Nagpal S, Deering DE (2010). Quantitative electroencephalographic abnormalities in fibromyalgia patients. Clin EEG Neurosci.

[29] Hämäläinen M, Hari R, Ilmoniemi RJ, Knuutila J, Lounasmaa OV (1993). Magnetoencephalography—theory, instrumentation, and application to noninvasive studies of the working human brain. Rev Mod Phys.

[30] Leiken KA, Xiang J, Curry E, Fujiwara H, Rose DF, Allen JR, Kacperski JE, O'Brien HL, Kabbouche MA, Powers SW, Hershey AD (2016). Quantitative neuromagnetic signatures of aberrant cortical excitability in pediatric chronic migraine. J Headache Pain.

[31] Ray NJ, Jenkinson N, Kringelbach ML, Hansen PC, Pereira EA, Brittain JS, Holland P, Holliday IE, Owen S, Stein J, Aziz T (2009). Abnormal thalamocortical dynamics may be altered by deep brain stimulation: using magnetoencephalography to study phantom limb pain. J Clin Neurosci.

[32] Walton KD, Dubois M, Llinas RR (2010). Abnormal thalamocortical activity in patients with complex regional pain syndrome (CRPS) type I. Pain.

[33] Lim M, Kim JS, Kim DJ, Chung CK (2016). Increased low- and high-frequency oscillatory activity in the prefrontal cortex of fibromyalgia patients. Front Hum Neurosci.

[34] Bennett RM, Friend R, Jones KD, Ward R, Han BK, Ross RL (2009). The revised fibromyalgia impact questionnaire (FIQR): validation and psychometric properties. Arthritis Res Ther.

[35] Zigmond AS, Snaith RP (1983). The hospital anxiety and depression scale. Acta Psychiatr Scand.

[36] Wolfe F, Smythe HA, Yunus MB, Bennett RM, Bombardier C, Goldenberg DL, Tugwell P, Campbell SM, Abeles M, Clark P (1990). The American College of Rheumatology 1990 criteria for the classification of fibromyalgia. Report of the multicenter criteria committee. Arthritis Rheum.

[37] Wolfe F (1997). The relation between tender points and fibromyalgia symptom variables: evidence that fibromyalgia is not a discrete disorder in the clinic. Ann Rheum Dis.

[38] Tadel F, Baillet S, Mosher JC, Pantazis D, Leahy RM (2011). Brainstorm: a user-friendly application for MEG/EEG analysis. Comput Intell Neurosci.

[39] Florin E, Baillet S (2015). The brain's resting-state activity is shaped by synchronized cross-frequency coupling of neural oscillations. NeuroImage.

[40] Hamalainen MS, Ilmoniemi RJ (1994). Interpreting magnetic fields of the brain: minimum norm estimates. Med Biol Eng Comput.

[41] Holmes CJ, Hoge R, Collins L, Woods R, Toga AW, Evans AC (1998). Enhancement of MR images using registration for signal averaging. J Comput Assist Tomogr.

[42] Klein A, Tourville J (2012). 101 labeled brain images and a consistent human cortical labeling protocol. Front Neurosci.

[43] Mantini D, Perrucci MG, Del Gratta C, Romani GL, Corbetta M (2007). Electrophysiological signatures of resting state networks in the human brain. Proc Natl Acad Sci U S A.

[44] Hsiao FJ, Cheng CH, Chen WT, Lin YY (2013). Neural correlates of somatosensory paired-pulse suppression: a MEG study using distributed source modeling and dynamic spectral power analysis. NeuroImage.

[45] Hsiao FJ, Yu HY, Chen WT, Kwan SY, Chen C, Yen DJ, Yiu CH, Shih YH, Lin YY (2015). Increased intrinsic connectivity of the default mode network in temporal lobe epilepsy: evidence from resting-state MEG recordings. PLoS One.

[46] Hsiao FJ, Chen WT, Wang PN, Cheng CH, Lin YY (2014). Temporo-frontal functional connectivity during auditory change detection is altered in Alzheimer's disease. Hum Brain Mapp.

[47] Nolte G, Bai O, Wheaton L, Mari Z, Vorbach S, Hallett M (2004). Identifying true brain interaction from EEG data using the imaginary part of coherency. Clin Neurophysiol.

[48] Guggisberg AG, Honma SM, Findlay AM, Dalal SS, Kirsch HE, Berger MS, Nagarajan SS (2008). Mapping functional connectivity in patients with brain lesions. Ann Neurol.

[49] Martino J, Honma SM, Findlay AM, Guggisberg AG, Owen JP, Kirsch HE, Berger MS, Nagarajan SS (2011). Resting functional connectivity in patients with brain tumors in eloquent areas. Ann Neurol.

[50] Hsiao FJ, Wang YJ, Yan SH, Chen WT, Lin YY (2013). Altered oscillation and synchronization of default-mode network activity in mild Alzheimer's disease compared to mild cognitive impairment: an electrophysiological study. PLoS One.

[51] Taylor KS, Seminowicz DA, Davis KD (2009). Two systems of resting state connectivity between the insula and cingulate cortex. Hum Brain Mapp.

[52] Fallon N, Alghamdi J, Chiu Y, Sluming V, Nurmikko T, Stancak A (2013). Structural alterations in brainstem of fibromyalgia syndrome patients correlate with sensitivity to mechanical pressure. Neuroimage Clin.

[53] Wood PB, Glabus MF, Simpson R, Patterson JC (2009). Changes in gray matter density in fibromyalgia: correlation with dopamine metabolism. J Pain.

[54] Cifre I, Sitges C, Fraiman D, Munoz MA, Balenzuela P, Gonzalez-Roldan A, Martinez-Jauand M, Birbaumer N, Chialvo DR, Montoya P (2012). Disrupted functional connectivity of the pain network in fibromyalgia. Psychosom Med.

[55] Jensen KB, Loitoile R, Kosek E, Petzke F, Carville S, Fransson P, Marcus H, Williams SC, Choy E, Mainguy Y, Vitton O, Gracely RH, Gollub R, Ingvar M, Kong J (2012). Patients with fibromyalgia display less functional connectivity in the brain's pain inhibitory network. Mol Pain.

[56] Cagnie B, Coppieters I, Denecker S, Six J, Danneels L, Meeus M (2014). Central sensitization in fibromyalgia? A systematic review on structural and functional brain MRI. Semin Arthritis Rheum.

[57] Brookes MJ, Hale JR, Zumer JM, Stevenson CM, Francis ST, Barnes GR, Owen JP, Morris PG, Nagarajan SS (2011). Measuring functional connectivity using MEG: methodology and comparison with fcMRI. NeuroImage.

[58] Attwell D, Iadecola C (2002). The neural basis of functional brain imaging signals. Trends Neurosci.

[59] Logothetis NK (2008). What we can do and what we cannot do with fMRI. Nature.

[60] Kitzbichler MG, Khan S, Ganesan S, Vangel MG, Herbert MR, Hamalainen MS, Kenet T (2015). Altered development and multifaceted band-specific abnormalities of resting state networks in autism. Biol Psychiatry.

[61] Houck JM, Cetin MS, Mayer AR, Bustillo JR, Stephen J, Aine C, Canive J, Perrone-Bizzozero N, Thoma RJ, Brookes MJ, Calhoun VD (2017). Magnetoencephalographic and functional MRI connectomics in schizophrenia via intra- and inter-network connectivity. NeuroImage.

[62] Kingsley JD (2012). Autonomic dysfunction in women with fibromyalgia. Arthritis Res Ther.

[63] Lerma C, Martinez A, Ruiz N, Vargas A, Infante O, Martinez-Lavin M (2011). Nocturnal heart rate variability parameters as potential fibromyalgia biomarker: correlation with symptoms severity. Arthritis Res Ther.

[64] Ploner M, Sorg C, Gross J (2017). Brain rhythms of pain. Trends Cogn Sci.

[65] Knyazev GG (2007). Motivation, emotion, and their inhibitory control mirrored in brain oscillations. Neurosci Biobehav Rev.

[66] Klimesch W (1999). EEG alpha and theta oscillations reflect cognitive and memory performance: a review and analysis. Brain Res Brain Res Rev.

[67] Taesler P, Rose M (2016). Prestimulus theta oscillations and connectivity modulate pain perception. J Neurosci.

[68] Ohara S, Crone NE, Weiss N, Lenz FA (2004). Attention to a painful cutaneous laser stimulus modulates electrocorticographic event-related desynchronization in humans. Clin Neurophysiol.

[69] Ohara S, Crone NE, Weiss N, Lenz FA (2006). Analysis of synchrony demonstrates ‘pain networks’ defined by rapidly switching, task-specific, functional connectivity between pain-related cortical structures. Pain.

[70] Liddle EB, Price D, Palaniyappan L, Brookes MJ, Robson SE, Hall EL, Morris PG, Liddle PF (2016). Abnormal salience signaling in schizophrenia: the role of integrative beta oscillations. Hum Brain Mapp.

[71] Knyazev GG (2011). Cross-frequency coupling of brain oscillations: an impact of state anxiety. Int J Psychophysiol.

[72] Meerwijk EL, Ford JM, Weiss SJ (2015). Resting-state EEG delta power is associated with psychological pain in adults with a history of depression. Biol Psychol.

[73] Styliadis C, Kartsidis P, Paraskevopoulos E, Ioannides AA, Bamidis PD (2015). Neuroplastic effects of combined computerized physical and cognitive training in elderly individuals at risk for dementia: an eLORETA controlled study on resting states. Neural Plast.

[74] Gram M, Graversen C, Olesen SS, Drewes AM (2015). Dynamic spectral indices of the electroencephalogram provide new insights into tonic pain. Clin Neurophysiol.

[75] Craig A, Tran Y, Wijesuriya N, Nguyen H (2012). Regional brain wave activity changes associated with fatigue. Psychophysiology.

[76] De Gennaro L, Marzano C, Veniero D, Moroni F, Fratello F, Curcio G, Ferrara M, Ferlazzo F, Novelli L, Concetta Pellicciari M, Bertini M, Rossini PM (2007). Neurophysiological correlates of sleepiness: a combined TMS and EEG study. NeuroImage.

[77] Legrain V, Iannetti GD, Plaghki L, Mouraux A (2011). The pain matrix reloaded: a salience detection system for the body. Prog Neurobiol.

