# Supplementary material for: Altered insula–default mode network connectivity in fibromyalgia: a resting-state magnetoencephalographic study
Source: J Headache Pain. 2017 Aug 23;18(1):89. doi: 10.1186/s10194-017-0799-x (PMC5567574; doi:10.1186/s10194-017-0799-x)
Supplement: Supplementary file 1 — Functional connectivity between the left insula and default mode network (12 cortical areas) in healthy controls and patients with fibromyalgia. (DOCX 20 kb) [file 10194_2017_799_MOESM1_ESM.docx]

Table S1 Functional connectivity between left insula and default mode network (12 cortical areas) in healthy control and fibromyalgia

|  |  | Delta | Theta | Alpha | Beta | Gamma |
| --- | --- | --- | --- | --- | --- | --- |
| Ins_L-IP_L | HC | 0.059±0.014 | 0.04±0.005 | 0.06±0.009 | 0.032±0.002 | 0.024±0.001 |
|  | FM | 0.048±0.006 | 0.035±0.004 | 0.059±0.008 | 0.028±0.003 | 0.020±0.001 |
| Ins_L-IP_R | HC | 0.061±0.015 | 0.048±0.008 | 0.058±0.008 | 0.024±0.002 | 0.022±0.001 |
|  | FM | 0.038±0.005 | 0.027±0.003 | 0.046±0.005 | 0.024±0.001 | 0.024±0.001 |
| Ins_L-MF_L | HC | 0.057±0.007 | 0.042±0.006 | 0.051±0.01 | 0.041±0.006 | 0.025±0.002 |
|  | FM | 0.056±0.014 | 0.037±0.004 | 0.036±0.005 | 0.031±0.002 | 0.022±0.001 |
| Ins_L-MF_R | HC | 0.068±0.014 | 0.045±0.006 | 0.051±0.008 | 0.040±0.006 | 0.024±0.002 |
|  | FM | 0.059±0.011 | 0.039±0.005 | 0.044±0.006 | 0.032±0.003 | 0.023±0.002 |
| Ins_L-MT_L | HC | 0.065±0.012 | 0.049±0.006 | 0.072±0.009 | 0.036±0.004 | 0.025±0.002 |
|  | FM | 0.054±0.011 | 0.045±0.006 | 0.064±0.007 | 0.032±0.003 | 0.023±0.002 |
| Ins_L-MT_R | HC | 0.065±0.013 | 0.048±0.006 | 0.071±0.01 | 0.037±0.005 | 0.023±0.002 |
|  | FM | 0.043±0.004 | 0.034±0.004 | 0.046±0.006 | 0.033±0.006 | 0.025±0.002 |
| Ins_L-PCu_L | HC | 0.057±0.01 | 0.033±0.007 | 0.045±0.006 | 0.031±0.005 | 0.022±0.002 |
|  | FM | 0.045±0.004 | 0.023±0.002 | 0.054±0.009 | 0.025±0.002 | 0.023±0.001 |
| Ins_L-PCu_R | HC | 0.049±0.006 | 0.038±0.004 | 0.049±0.005 | 0.033±0.004 | 0.024±0.001 |
|  | FM | 0.061±0.011 | 0.025±0.003 | 0.050±0.007 | 0.028±0.002 | 0.023±0.002 |
| Ins_L-PCC_L | HC | 0.058±0.009 | 0.042±0.005 | 0.041±0.005 | 0.036±0.006 | 0.024±0.001 |
|  | FM | 0.048±0.005 | 0.030±0.002 | 0.047±0.005 | 0.026±0.002 | 0.022±0.001 |
| Ins_L-PCC_R | HC | 0.062±0.012 | 0.045±0.006 | 0.050±0.007 | 0.037±0.006 | 0.024±0.002 |
|  | FM | 0.054±0.007 | 0.027±0.003 | 0.044±0.005 | 0.026±0.002 | 0.022±0.001 |
| Ins_L-LT_L | HC | 0.072±0.019 | 0.041±0.007 | 0.046±0.006 | 0.034±0.003 | 0.021±0.001 |
|  | FM | 0.048±0.006 | 0.029±0.004 | 0.040±0.006 | 0.028±0.003 | 0.024±0.001 |
| Ins_L-LT_R | HC | 0.059±0.008 | 0.042±0.006 | 0.046±0.006 | 0.029±0.002 | 0.024±0.001 |
|  | FM | 0.045±0.005 | 0.033±0.003 | 0.048±0.005 | 0.026±0.002 | 0.022±0.001 |

L, left; R, right; Ins, insula; IP, inferior parietal cortex; MF, medial frontal cortex; MT,

medial temporal cortex; PCu, precuneus; PCC, posterior cingulate cortex; LT, lateral temporal regions.
